# UVH6 regulates osmotic and heat stress tolerance by modulating transcription

**DOI:** 10.3389/fpls.2025.1623563

**Published:** 2025-09-18

**Authors:** Koya Kobayashi, Yusuke Murakoshi, Kazuki Kanamori, Jun Hidema, Goro Masuda, Keisuke Tanaka, Izumi Yotsui, Yoichi Sakata, Teruaki Taji

**Affiliations:** ^1^ Department of Bioscience, Tokyo University of Agriculture, Tokyo, Japan; ^2^ Research Center for Space Agriculture and Horticulture, Chiba University, Chiba, Japan; ^3^ NODAI Genome Center, Tokyo University of Agriculture, Tokyo, Japan

**Keywords:** osmotic stress response, heat stress response, DNA damage, transcription, Arabidopsis thaliana accession

## Abstract

Nucleotide excision repair (NER) is a critical mechanism for repairing DNA damage, including UV-induced lesions and chemically induced adducts. The *UVH6* gene encodes a subunit of the transcription factor IIH complex and is essential for both NER and transcription initiation. In *Arabidopsis thaliana*, *UVH6* mutations impair DNA repair, enhance UV sensitivity, and decrease heat stress tolerance. We here isolated *acquired osmotolerance–defective12* (*aod12*) mutant derived from osmotolerant Bu-5 accession; this mutant had pale green leaves and was osmosensitive and heat sensitive. Genetic and molecular analyses revealed that a mutation in *UVH6* underlies these phenotypes of *aod12*. RNA sequencing demonstrated that *UVH6* is necessary for appropriate transcriptional responses under osmotic stress, as expression of some stress-response genes was altered in *aod12*. Expression of pathogenesis-related genes and cell death were increased, indicating that immune responses detrimental to osmotolerance were activated. Interestingly, UVH6-mediated osmotolerance was independent of its canonical DNA repair function, as other NER-related mutants (*xpf*, *xpg*, *ercc1*) were not osmosensitive. Signaling pathways involving UVR8 and SOG1 were not implicated in *UVH6* mutation–induced immune responses, suggesting a novel regulatory mechanism linking transcriptional control and stress tolerance. This study highlights UVH6 as a key integrator of genome stability, transcription, and stress resilience in plants.

## 1 Introduction

Nucleotide excision repair (NER) is a vital mechanism for repairing DNA damage that distorts the double-helix structure, including UV-induced cyclobutane pyrimidine dimers (CPDs), pyrimidine (6-4) pyrimidinone dimers (6–4 photoproducts), and bulky base adducts caused by chemicals such as cisplatin (Kunz et al., 2005; Marteijn et al., 2014). The NER pathway is highly conserved from *Escherichia coli* to humans (Marteijn et al., 2014). In *Arabidopsis thaliana* (Arabidopsis), the NER pathway involves several protein heterodimers that function sequentially in lesion recognition, DNA unwinding, and damage-induced incisions at the 5′ and 3′ ends of the damaged DNA strand. Initially, the heterodimer of XPC and RAD4 detects structural distortions in the DNA helix caused by damage. Then the helicase activity of the XPB1 and XPD (UVH6) heterodimer separates the double-stranded DNA around the lesion to create an open complex to allow access of repair proteins. Finally, the 5′ end of the damaged strand is cleaved by the endonuclease heterodimer XPF and RAD1, while the 3′ end is cleaved by ERCC1 and XPG. These coordinated activities ensure the precise removal of the damaged DNA fragment, allowing subsequent repair synthesis and ligation to restore the integrity of the genome.

UVH6 is the Arabidopsis ortholog of the human XPD and yeast RAD3 proteins and functions as part of the TFIIH complex, which is involved in transcription initiation and NER (Liu et al., 2003; Vonarx et al., 2006). The *uvh6* mutant was initially identified as hypersensitive to UV-B and UV-C radiation in Arabidopsis (Jenkins et al., 1995, Jenkins et al., 1997). Several *uvh6* mutant alleles have been isolated, including *uvh6–1* and *uvh6-4*, which lead to growth defects and pale green leaves because of low chlorophyll content (Bourguet et al., 2018). The inability to recover a homozygous *uvh6–2* mutant, presumed to have non-functional *UVH6* due to a T-DNA insertion, suggests that UVH6 is essential for viability (Liu et al., 2003; Bourguet et al., 2018). The *uvh6* mutants are hypersensitive not only to UV radiation stress but also to heat, cold, and freezing temperatures (Jenkins et al., 1995; Hall et al., 2009). In Arabidopsis, UVH6 and MED14, a subunit of the MEDIATOR complex involved in transcription initiation, are required for heat stress–induced transcriptional changes and the release of heterochromatin silencing (Bourguet et al., 2018). Under heat stress, the expression of more than 4,700 genes and 230 transposable elements is lower in *uvh6–3* mutants than in the wild type (WT), demonstrating that UVH6 is necessary for efficient genome-wide transcription under heat stress (Bourguet et al., 2018). Collectively, UVH6 plays a dual role in NER and transcription regulation; however, its importance for osmotic and heat stress tolerance remains unclear.

Osmotic stress, caused by drought, salinity, or low temperatures, inhibits plant growth by inducing the overproduction of reactive oxygen species, which cause oxidative damage, DNA lesions, and ultimately cell death (Imlay and Linn, 1988; Sharma et al., 2012). One key priming effect observed in response to osmotic stress is acquired osmotolerance: mild stress exposure helps plants to acquire tolerance and to withstand more severe stress (Sung et al., 2003). Many Arabidopsis accessions acquire osmotolerance after mild salt stress (Katori et al., 2010). Exposing 7-day-old seedlings to 100 mM NaCl for 7 days (acclimation period) induces tolerance to 750 mM sorbitol in some Arabidopsis accessions, even though they cannot survive at 650 mM sorbitol without prior salt acclimation (Katori et al., 2010).

We have identified a nucleotide-binding leucine-rich repeat gene, *ACQUIRED OSMOTOLERANCE* (*ACQOS*), which plays a role in osmotolerance (Ariga et al., 2017). In the absence of osmotic stress, this gene contributes to antibacterial resistance, but under osmotic stress it triggers autoimmunity and compromises osmotolerance. Such autoimmunity leads to programmed cell death. Osmotolerance is lower in accessions with functional *ACQOS* alleles (e.g., Col-0), than in those with non-functional alleles (e.g., Zu-0 and Bu-5).

To identify the genetic factors underlying acquired osmotolerance, we have conducted a forward genetic screen for mutants with *acquired osmotolerance–defective* (*aod*) phenotypes using ion-beam-mutagenized Bu-5 seeds. Among the identified mutants, *aod2* carries a mutation in the *ECERIFERUM 10* (*CER10*) gene, which is involved in very long-chain fatty acid elongation for cuticular wax synthesis (Fukuda et al., 2022); *aod1* has a mutation in *CONSTITUTIVELY ACTIVATED CELL DEATH 1* (*CAD1*) (Murakoshi et al., 2024), *aod6* in *CATION CALCIUM EXCHANGER4* (*CCX4*) (Kanamori et al., 2023), and *aod13* in *MAP KINASE PHOSPHATASE1* (*MKP1*) (Uchida et al., 2022). These results highlight the roles of cuticular wax biosynthesis, calcium transport via CCX4, and immune suppression mediated by MPK3/6 dephosphorylation in acquired osmotolerance. Immune responses of the *aod1*, *aod6*, and *aod13* mutants are enhanced under osmotic stress, emphasizing the critical role of immune suppression in osmotolerance (Uchida et al., 2022; Kanamori et al., 2023; Murakoshi et al., 2024).

Here, we identified the *aod12* mutant and its causative gene involved in osmotolerance. We hypothesized that the loss of osmotic and heat tolerance in *aod12* is not caused by a defect in its canonical DNA repair function but rather by a defect in its unique transcriptional regulatory function. By uncovering the regulatory mechanisms of the *aod12* mutant, we aim to advance our understanding of how UVH6 contributes to osmotic stress responses in Arabidopsis.

## 2 Materials and methods

### 2.1 Plant materials and growth conditions

*Arabidopsis thaliana* seeds (Bu-5 or Col-0) were sown on agar (0.8% w/v) plates containing full-strength Murashige and Skoog (MS) salts with a vitamin mixture (10 mg L^−1^ myoinositol, 200 μg L^−1^ glycine, 50 μg L^−1^ nicotinic acid, 50 μg L^−1^ pyridoxine hydrochloride, 10 μg L^−1^ thiamine hydrochloride, pH 5.7), and 1% w/w sucrose. Plates were sealed with surgical tape, the seeds were stratified at 4°C for 4–7 days and transferred to a growth chamber (80 μmol photons m^2^ s^−1^; 16/8-h light/dark cycle; 22°C) for germination and growth.

Bu-5 seeds were irradiated as described in (Uchida et al., 2022).

Seeds of the following Arabidopsis mutants in the Col-0 background were obtained from the Arabidopsis Biological Resource Center (Ohio State University): *uvh6-1* (CS6375), *xpf* (SALK096156C), *xpg* (CS3820), and *ercc1* (SALK077000C). Seeds of the *sog1–1* mutant (Col-0 background) were kindly provided by Dr. Kaoru Yoshiyama of Tohoku University. To generate a *uvh6–1 sog1–1* plant, we crossed a *uvh6–1* plant with the *sog1–1* mutant.

### 2.2 Stress treatment for the acquired osmotolerance assay

Seedlings were grown on nylon mesh (990 μm) on an MS agar plate. At 7 days of age, they were mesh-transferred to a plate supplemented with 100 mM NaCl for 7 days. The seedlings were then mesh-transferred to a plate supplemented with 750 mM sorbitol for 36–49 days. Aerial parts of six randomly chosen seedlings from each experimental group (with or without various stresses) were harvested and subsequently homogenized in cold acetone. Chlorophyll content was determined spectrophotometrically using the equations described in (Porra et al., 1989).

### 2.3 Abiotic stress assays

All abiotic stress assays were performed in a growth chamber at 22 °C under a 16/8-h light/dark cycle with a light intensity of 80 μmol photons m^-^² s^-^¹. Seedlings were grown on nylon mesh (990 μm) on an MS agar plate. At 10 days of age, they were mesh-transferred to a plate supplemented with 650 mM sorbitol for 31 days (osmotic-shock stress) or 5 μM paraquat for 14 days (oxidative stress). Chlorophyll content was determined as described in (Porra et al., 1989).

### 2.4 RNA extraction and qRT-PCR

Total RNA extraction and qRT-PCR were performed as described in (Isono et al., 2023). *ACTIN2* was used as an internal standard for qRT-PCR. The PCR primers are listed in **Supplementary Table 2**.

### 2.5 Genetic mapping of the causative gene of *aod12*

We crossed the *aod12* mutant with Pog-0, an accession that shows acquired osmotic stress tolerance, and selfed the resulting F_1_ progeny to generate an F_2_ population. Genomic DNA was prepared from individual F_2_ plants with the recessive phenotype for use as PCR templates. We used the simple sequence-length polymorphism (SSLP) markers listed in **Supplementary Table 2** for mapping. PCR conditions were as follows: initial denaturation at 94 °C for 2 min; 34 cycles at 94 °C for 20 s, 52–55 °C for 20 s, and 72 °C for 20 s; and final extension at 72 °C for 2 min. The microsatellites were fractionated in 5%–7% agarose gels, and the recombination frequencies (%) were calculated from the band pattern.

### 2.6 DNA library construction and sequencing

Mutations were detected in the whole-genome sequencing data of *aod12* as described in (Uchida et al., 2022). RNA-seq analysis was performed with three biological replicates for each condition. Differentially expressed genes (DEGs) were identified based on the criteria for fold change and FDR. The read data and the RNA-seq data were submitted to the DNA Data Bank of Japan (DDBJ) Read Archive (acc. Nos. DRR641246 and DRR641293–DRR641304).

### 2.7 Plasmid construction and transformation

For complementation analysis, we amplified the genomic region of *AOD12/UVH6* (2.0-kb upstream of the ATG initiation codon) by PCR with the pRI909 UVH6 F and pRI909 UVH6 R primers (**Supplementary Table 2**) and cloned it into the pRI909 vector (Takara Bio Inc.). The construct was introduced into *Agrobacterium tumefaciens* strain GV3101. Plants were transformed with the agrobacterium using the floral dip method. Transgenic plants were selected on MS agar plates containing 200 µg mL^−1^ claforan and 20 µg mL^−1^ hygromycin. Ten-day-old seedlings (T_1_ plants) were transferred into soil pots.

### 2.8 DNA damage assay

The DNA damage assay was performed under the same growth chamber conditions as the abiotic stress assays, which were 22 °C with a 16/8-h light/dark cycle and a light intensity of 80 μmol photons m^-^² s^-^¹. To determine single-strand break (SSB) frequency, DNA extracted from seedlings was denatured by adding alkaline solution (0.5 M NaOH, 10% glycerol, and 0.25% [w/v] bromocresol green) and incubating for 30 min at 37 °C. DNA molecules were separated according to their single-strand molecular lengths in 0.7% alkaline agarose gels using static field electrophoresis and biased sinusoidal field gel electrophoresis (Genofield; ATTO Co.) (Hidema and Kumagai, 1998).

To determine double-strand break (DSB) frequency, DNA extracted from seedlings was mixed with loading buffer (10% glycerol and 0.25% [w/v] bromocresol green in TE buffer). DNA molecules were separated according to their single-strand molecular lengths in TBE agarose gels using static field electrophoresis and biased sinusoidal field gel electrophoresis (Genofield) (Hidema and Kumagai, 1998). The molecular length markers were DNA from *Hansenula wingei* chromosomes (smallest, 1.05 Mb) (Bio-Rad), bacteriophage T4 (170 kb), bacteriophage λ (48.5 kb), and the HindIII digest of λ DNA (23.1, 9.4, 6.6, 4.3, and 2.3 kb).

The SSB or DSB frequency was determined using a DNA damage analysis system (Tohoku Electric Co.) as previously described (Hidema and Kumagai, 1998) from a molecular length standard curve. DNA at each migration position was quantified and the values were expressed as SSB or DSB per 10^6^ bp.

### 2.9 Trypan blue staining

Cell death was detected by trypan blue staining as previously described (Tsukimoto et al., 2021).

### 2.10 Statistical analysis

Statistical significance analysis was performed using the Student’s *t*-test. The significance results are indicated by asterisks: *P<0.05, **P<0.01, and ***P<0.001. For multiple comparisons, one-way ANOVA with *post hoc* Tukey HSD test was conducted. The 0.05 level of probability was used as the criterion for significance.

### 2.11 UVH6 structure prediction and visualization

UVH6 protein structure predictions were performed using AlphaFold3 (Abramson et al., 2024) via the AlphaFold Server (https://alphafoldserver.com/welcome). For the superimposition of *Arabidopsis thaliana* XPD/UVH6 with human XPD and for domain-specific coloring, PyMOL Molecular Graphics System, Version 3.0 (Schrödinger, LLC.) was utilized. The positions of the domains were determined by aligning UVH6 with XPD and referencing the positions indicated in Peissert et al., 2020 (Peissert et al., 2020).

## 3 Results

### 3.1 Identification and characterization of *aod12*, a mutant impaired in acquired osmotolerance and heat stress tolerance

**Figure 1:**
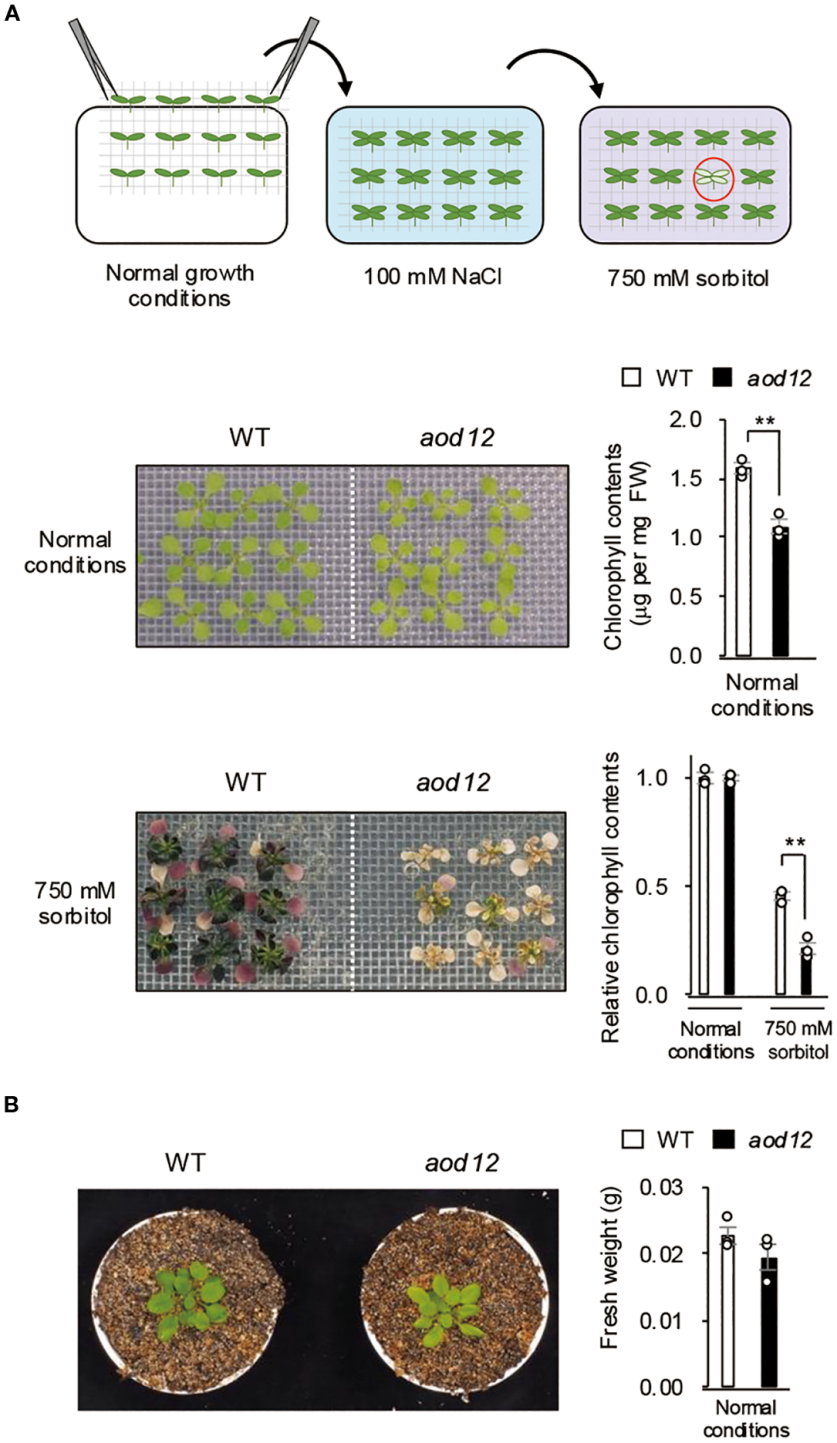
Identification of the *acquired osmotolerance–defective 12* (*aod12*) mutant. **(A)** Upper panel: flow chart of the acquired osmotolerance assay. Salt-acclimated 2-week-old seedlings of accession Bu-5 were mesh-transferred to Murashige and Skoog (MS) agar plates containing 750 mM sorbitol for 49 days. Hypersensitive seedlings (red circle) were selected as *aod* mutants. Middle panel: images of 2-week-old wild-type (WT) and *aod12* seedlings grown under normal conditions and their chlorophyll content; FW, fresh weight. Lower panel: images of WT and *aod12* seedlings subjected to acquired osmotolerance assay and chlorophyll content under these conditions relative to that under normal conditions. For each group, chlorophyll was quantified from the aerial parts of six randomly chosen seedlings (mean ± SE, *n* = 3). **P < 0.01 (Student’s *t*-test). **(B)** Left panel: Representative 5-week-old WT and *aod12* plants grown in soil under normal conditions. Right panel: Fresh weight of 17-day-old seedlings grown on MS medium under normal conditions. Data represent means ± SE (n=3 biological replicates).

To identify genes involved in acquired osmotolerance, we screened over 30,000 ion-beam-mutagenized M2 (second generation after mutagenesis) seed pools derived from the osmotolerant Bu-5 accession and isolated the mutant *aod12*. Under normal growth conditions, *aod12* leaves were pale green and chlorophyll content was significantly lower than in the WT (**Figure 1A**). Therefore, the osmotolerance of their seedlings was evaluated on the basis of chlorophyll content relative to that under normal conditions. After exposure to 100 mM NaCl, acquired osmotolerance to 750 mM sorbitol was significantly lower in *aod12* seedlings than in WT Bu-5 (**Figure 1A**). Under normal growth conditions, 17-day-old *aod12* and WT seedlings exhibited similar fresh weights (**Figure 1B**). While grown in soil for 5 weeks, *aod12* retained its characteristic pale-green rosette leaves, a phenotype consistent with earlier observations (**Figure 1B**).

**Figure 2:**
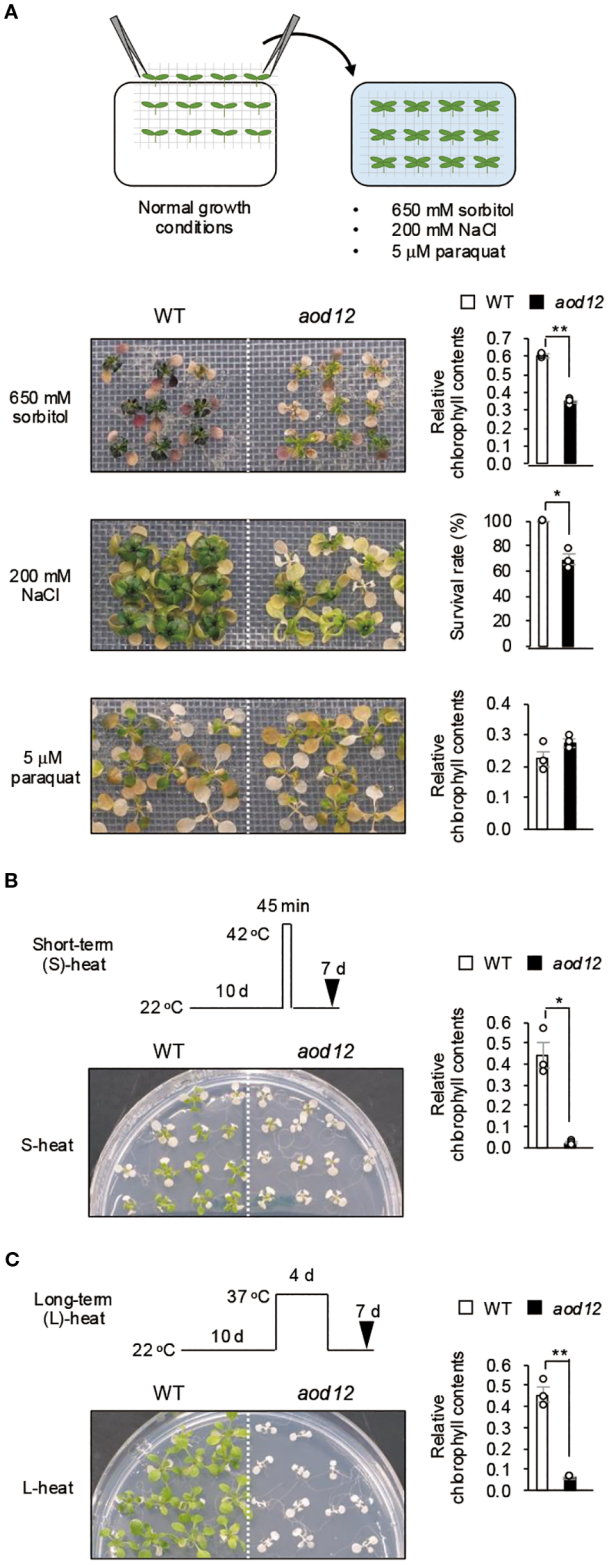
Tolerance of *aod12* to abiotic stresses. **(A)** Upper panel: flow chart of the osmotic- and salt-shock and oxidative-stress tolerance assays. Lower panels: plant images (left) and quantification of tolerance (right). Ten-day-old seedlings were mesh-transferred to MS agar plates containing 650 mM sorbitol for 31 days (top) or 5 μM paraquat (an inducer of oxidative stress) for 14 days (bottom). For each group, chlorophyll was quantified from the aerial parts of six randomly chosen seedlings. Chlorophyll content under stress relative to that under normal conditions (for osmotic and oxidative stress) was used to assess stress tolerance. **(B, C)** Tolerance of *aod12* to **(B)** S-heat stress and **(C)** L-heat stress. Ten-day-old WT and *aod12* seedlings grown at 22 °C (normal conditions) were placed **(B)** at 42 °C for 45 min or **(C)** at 37 °C for 4 days and returned to 22 °C for 7 days. Plant images (left) and chlorophyll content under heat stress relative to that under normal conditions (right).(**D**) Expression of *HSP70* and *HSP17.6* in *aod12*. Expression of *HSP70* and *HSP17.6* under normal and heat-stress conditions; expression relative to *ACTIN2* was determined by qRT-PCR. Ten-day-old seedlings grown at 22 °C (0 h) were placed at 37 °C for1 or 24 **(h)** In all panels, bar graphs show mean ± SE (*n* = 3); *P < 0.05, **P < 0.01 (Student’s *t*-test).

To determine whether the *aod12* phenotype was broadly tolerant to stresses, we assessed its tolerance to other abiotic stresses, namely osmo-shock, salt shock, oxidative stress (paraquat) (**Figure 2A**), short-term heat (S-heat) stress (**Figure 2B**), and long-term heat (L-heat) stress (**Figure 2C**). The *aod12* mutant showed significantly reduced tolerance to osmotic shock, S-heat, and L-heat stresses compared to WT Bu-5, but no difference in oxidative stress tolerance.

Furthermore, to gain insight into the molecular basis of the observed heat sensitivity in *aod12*, we examined the expression of *heat shock protein* (*HSP*) genes under heat stress. Our analysis revealed that the expression levels of both *HSP70* and *HSP17.6* were significantly lower in *aod12* compared to WT when subjected to 37 °C for 1 hour (**Figure 2D**). This attenuated *HSP* induction is consistent with previous findings of a genome-wide reduction in gene expression in *aod12* under heat stress conditions (Bourguet et al., 2018). These results suggest that the hypersensitivity of *aod12* to heat stress is likely due to the impaired induction of essential stress-responsive genes, including *HSP*s, which are critical for heat tolerance.

### 3.2 *UVH6* is the causal gene for osmosensitivity in *aod12*

**Figure 3:**
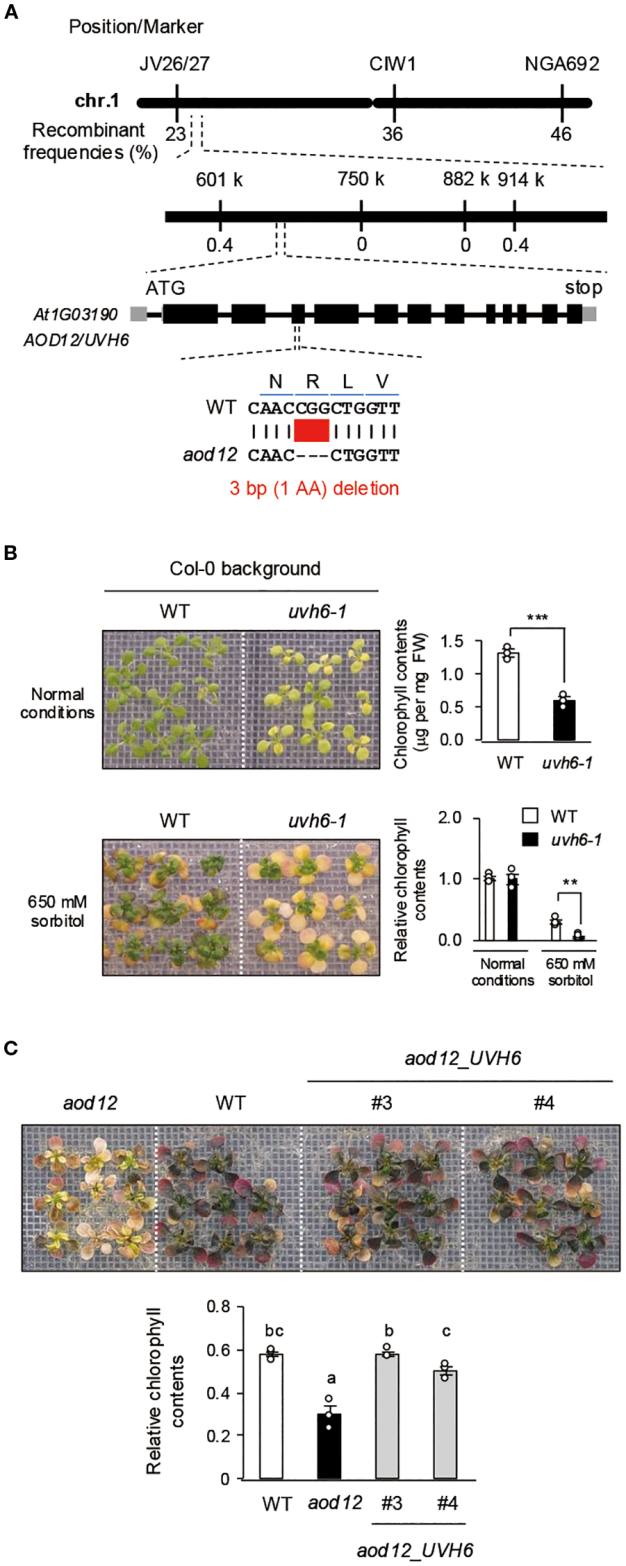
Identification of the causal gene *UVH6* (*At1G03190*) in *aod12*. **(A)** High-resolution mapping of the causal locus in *aod12.* F_2_ plants from *aod12* × Pog-0 were used for mapping. The causal codon deletion is shown in red. **(B, C)** Acquired osmotolerance of **B**) WT and the *uvh6–1* mutant and **C**) WT, *aod12*, and complementation lines (*aod12*_*UVH6*). Salt-acclimated 2-week-old seedlings were mesh-transferred to MS agar plates containing 650 mM sorbitol for 39 days. Plant images and the corresponding chlorophyll contents are shown; FW, fresh weight. For each group, chlorophyll was quantified from the aerial parts of six randomly chosen seedlings. Relative chlorophyll content under stress was calculated by normalizing to that under normal growth conditions. Data represent means ± SE from three biological replicates (n = 3). In **B**), **P < 0.01, ***P < 0.001 (Student’s *t*-test). In **C**), the same letters indicate no significant difference (*P* < 0.05, one-way ANOVA with *post hoc* Tukey HSD test).

To identify the locus responsible for the osmosensitive phenotype of *aod12*, we crossed it with Pog-0, an accession with acquired osmotolerance (Ariga et al., 2017), and performed high-resolution chromosomal mapping. The locus was mapped to a 313-kbp region on chromosome 1 (**Figure 3A**). Whole-genome sequencing of *aod12* and WT Bu-5 revealed a 3-bp deletion in *At1g03190*, causing a deletion of a single arginine in the UVH6 protein (296) in *aod12* (**Figure 3A**). *At1g03190* was previously identified as responsible for the *uvh6* mutant, which is hypersensitive to UV. To confirm that *UVH6* is the causal gene of *aod12*, we evaluated the acquired osmotolerance of the mutant *uvh6-1* (Col-0 background strong allele); (Liu et al., 2003). Since Col-0 lacks acquired osmotolerance, we acclimated the seedlings with 100 mM NaCl and exposed them to 650 mM sorbitol. The *uvh6–1* mutant had pale green leaves under normal conditions and an osmosensitive phenotype, as did *aod12* (**Figure 3B**). Complementation of *aod12* with *UVH6* with its native promoter (*aod12_UVH6*) restored acquired osmotolerance and leaf color to Bu-5 WT levels (**Figure 3C**, **Supplementary Figure 1**). Therefore, *UVH6* is the causal gene for the osmosensitive and pale-green-leaf phenotype of *aod12*.

### 3.3 *UVH6* mutation impairs osmotolerance independently of DNA repair

**Figure 4:**
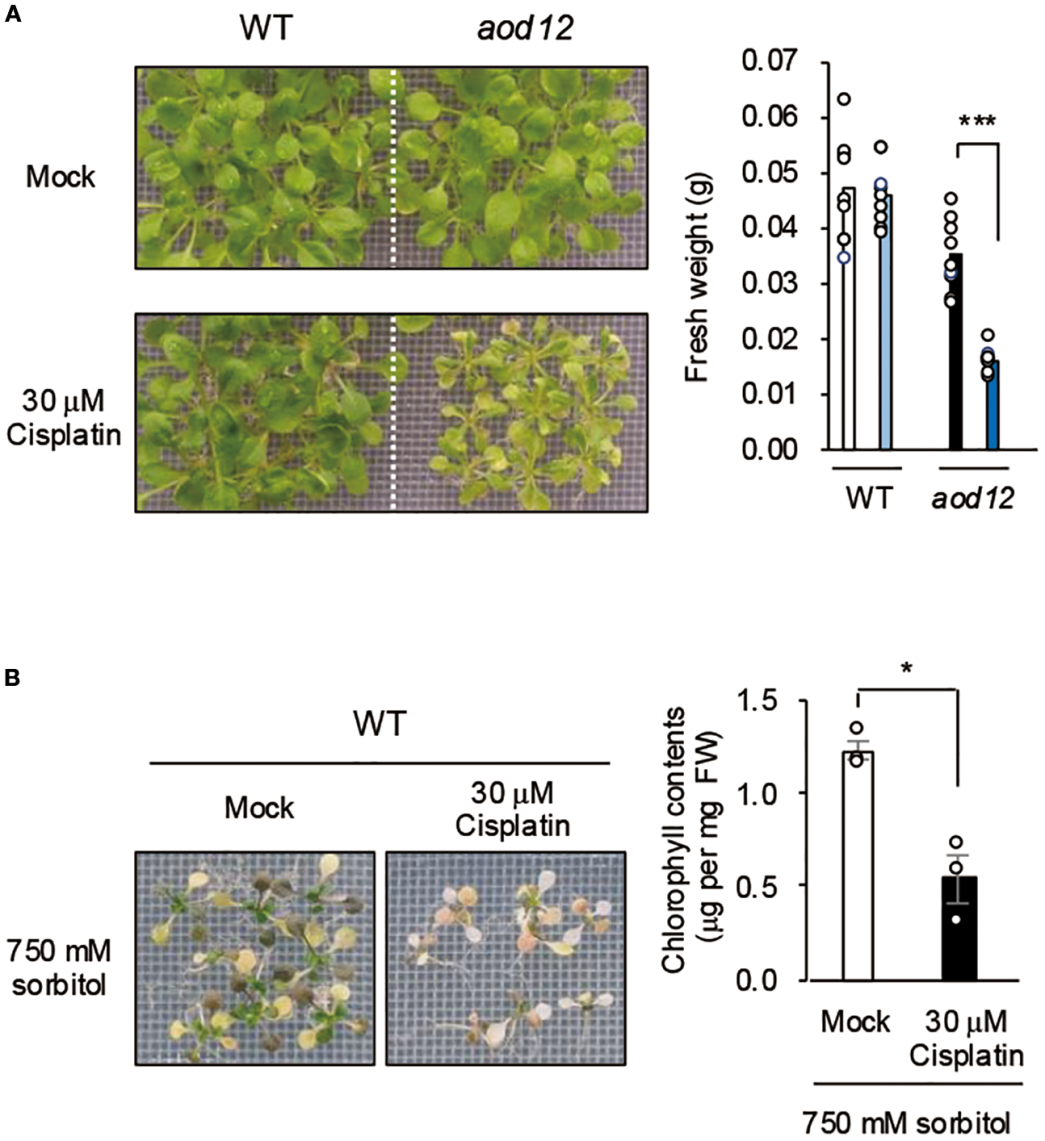
Effects of DNA damage on osmotolerance. **(A)** Effect of cisplatin treatment on growth of WT Bu-5 and *aod12*. **(B)** Effect of cisplatin treatment on osmotolerance of WT. Ten-day-old WT seedlings were mesh-transferred to MS agar plates containing 30 μM cisplatin for 6 h, and then mesh-transferred to **A**) MS agar plates for 7 days or **B**) MS agar plates containing 650 mM sorbitol for 27 days. Images of plants and bar graphs for their chlorophyl content are shown. For each group, chlorophyll was quantified from the aerial parts of six randomly chosen seedlings. Blue, cisplatin treatment. In both panels, the bar graphs show mean ± SE from three biological replicates (n = 3); *P < 0.05, ***P < 0.001 (Student’s *t*-test).

UVH6 is a component of the TFIIH complex, which has two main functions: facilitating transcription initiation by RNA polymerase II and participating in NER, a key DNA repair pathway (Kciuk et al., 2020). NER repairs DNA damage spanning multiple bases, which is often caused by cross-linked structures (Kciuk et al., 2020). To assess *aod12* sensitivity to DNA damage stress, we used cisplatin, which induces cross-linked DNA structures and inhibits growth. The sensitivity to cisplatin was higher in the *aod12* mutant than in WT (**Figure 4A**), suggesting reduced DNA repair in *aod12*. This interpretation is strongly supported by prior studies in Arabidopsis demonstrating that the NER pathway is crucial for repairing cisplatin-induced DNA damage. Specifically, *in vitro* repair synthesis assays have shown that the repair of cisplatin-damaged plasmid DNA is significantly reduced in plants with depleted AtRAD1/ERCC1 activity, a likely homolog of mammalian ERCC1, a key NER component (Li et al., 2002). To evaluate the effect of DNA damage on osmotic stress tolerance, we treated WT seedlings with mock or cisplatin for 6 h and then exposed them to osmotic stress. Sensitivity to osmotic stress was higher in cisplatin-treated plants than in mock-treated plants (**Figure 4B**), indicating that DNA repair plays a role in the osmotic stress response in Arabidopsis.

**Figure 5:**
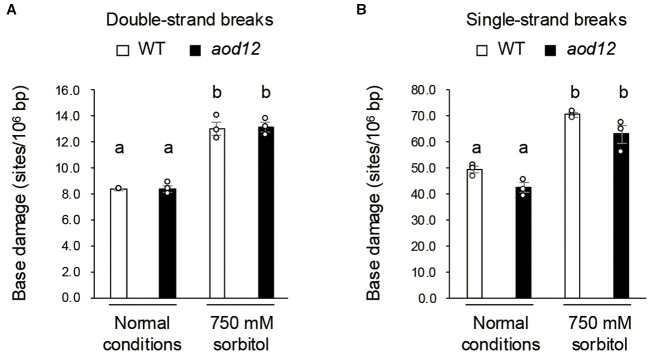
DNA damage under osmotic stress. **(A)** Double-strand breaks. **(B)** Single-strand breaks. Two-week-old seedlings were grown on MS (normal conditions) or salt-acclimated 2-week-old seedlings were mesh-transferred to MS agar plates containing 750 mM sorbitol for 10 days (osmotic stress). Data represent means ± SE from three biological replicates (n = 3). The same letters above the bars indicate no significant difference (*P* < 0.05, one-way ANOVA with *post hoc* Tukey HSD test).

**Figure 6:**
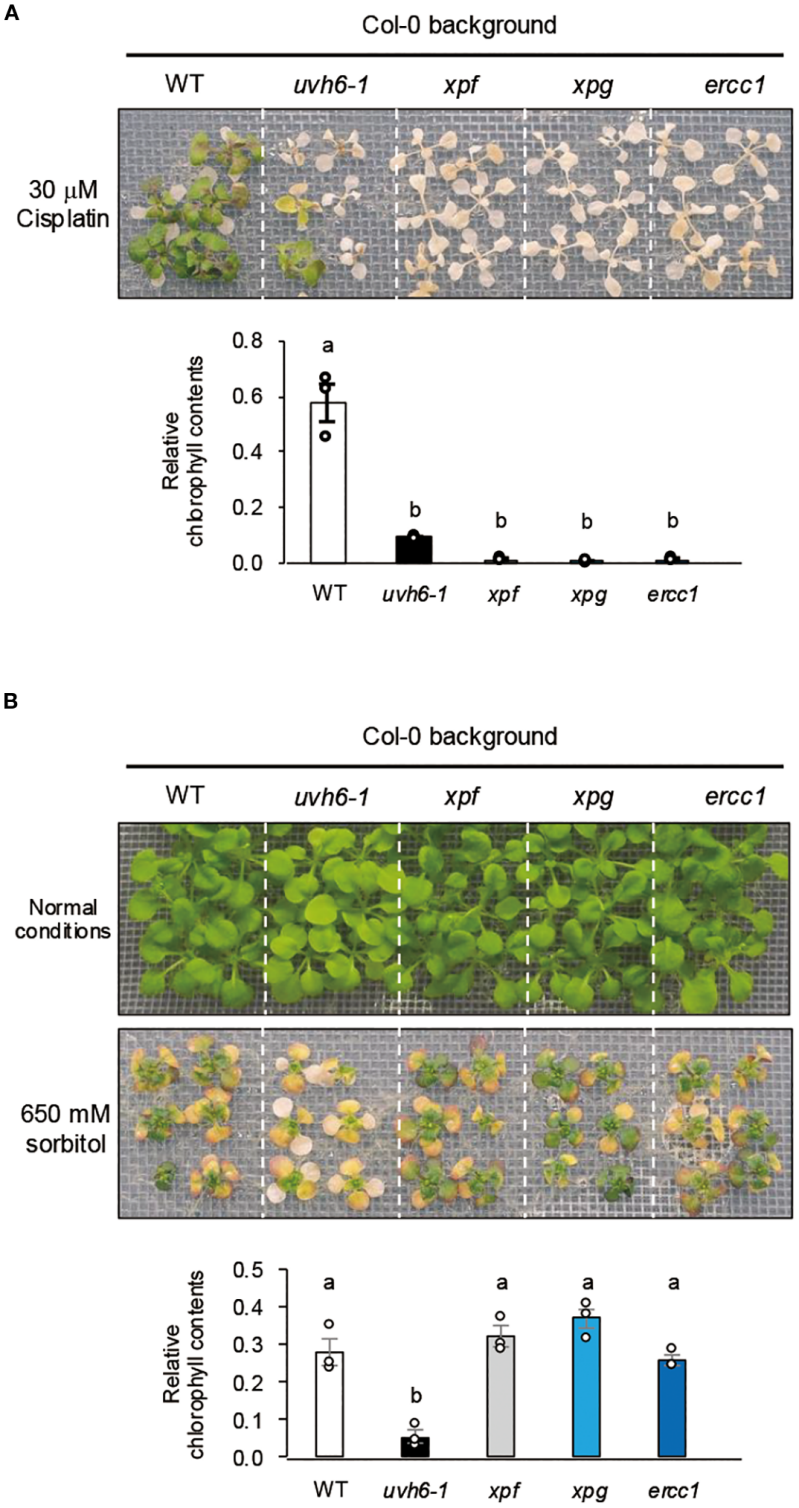
Cisplatin sensitivity and acquired osmotolerance of NER-related mutants. **(A)** Cisplatin sensitivity. Ten-day-old seedlings were mesh-transferred to MS agar plates containing 30 μM cisplatin for 6 h, and then mesh-transferred to MS agar plates for 13 days. **(B)** Acquired osmotolerance. Salt-acclimated 2-week-old seedlings were mesh-transferred to MS agar plates containing 650 mM sorbitol for 13 days. Images: osmotolerance of WT Col-0 and the indicated mutants. Bar graph shows chlorophyll content under osmotic stress relative to that under normal conditions. For each group, chlorophyll was quantified from the aerial parts of six randomly chosen seedlings (mean ± SE, *n* = 3). The same letters above the bars indicate no significant difference (*P* < 0.05, one-way ANOVA with *post hoc* Tukey HSD test).

Next, we investigated whether osmotic stress causes double- or single-strand DNA breaks and whether DNA damage accumulation differs between WT and *aod12*. Salt-acclimated seedlings were exposed to 750 mM sorbitol for 10 days, and the accumulation of DNA breaks was monitored. Osmotic stress induced considerable DNA damage, but no significant differences were observed between WT and *aod12* (**Figure 6**). These findings suggest that, although the *UVH6* mutation in *aod12* impairs DNA repair, it does not significantly affect the repair of osmotic stress–induced DNA damage.

**Figure 7:**
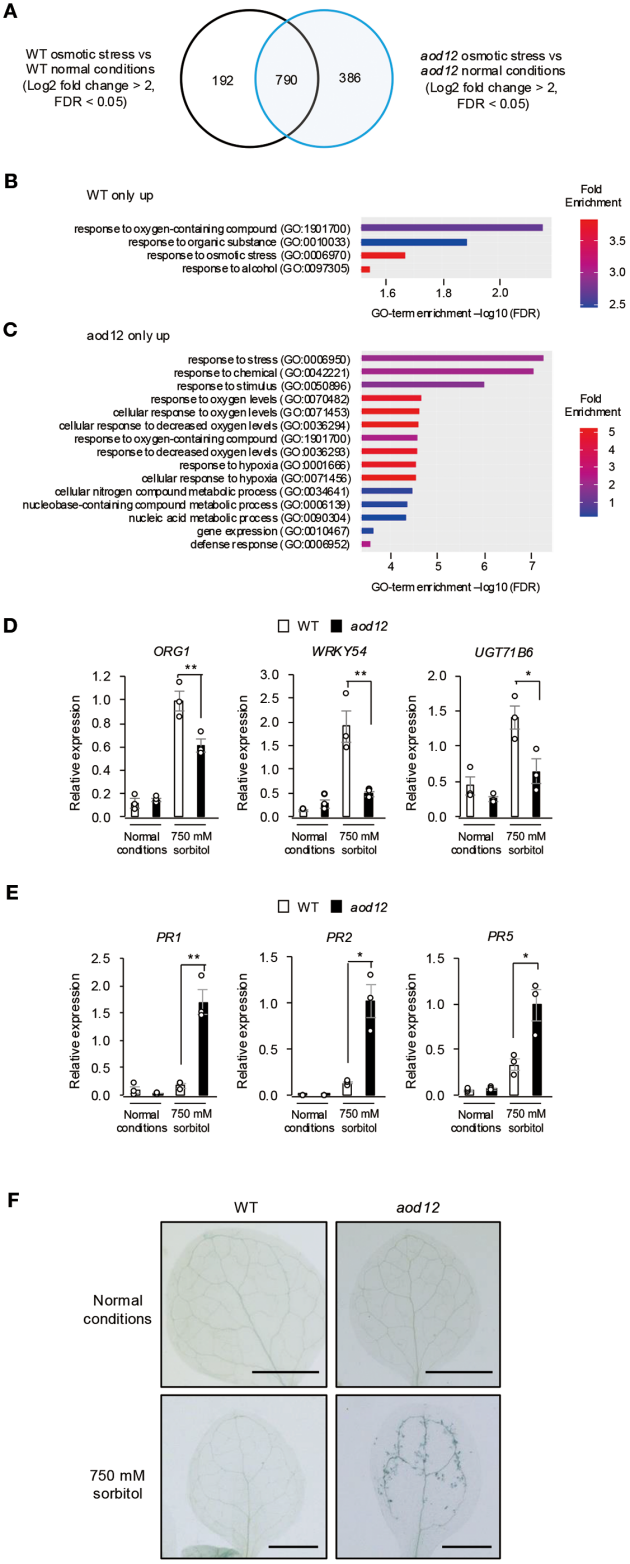
Effect of osmotic stress on gene expression in *aod12*. **(A)** Venn diagram of the number of differentially expressed genes (DEGs) between normal conditions and osmotic stress (100 mM NaCl for 7 days followed by 750 mM sorbitol for 8 h) in WT Bu-5 and *aod12*. **(B, C)** GO enrichment analysis of **(B)** 192 specific DEGs in WT and **(C)** 386 specific DEGs in *aod12* for top 15 biological processes based on positive fold enrichment. DEGs were classified according to the GO terms in Geneontology software (https://geneontology.org/) into categories based on biological processes. **(D, E)** Expression profiles of **(D)** osmotic stress–responsive genes and **(E)** PR genes in WT and *aod12* seedlings under normal conditions and osmotic stress applied as in **(A)** for **(D)** 8 h or **(E)** 3 days; transcript levels were determined by quantitative real-time PCR relative to those of *ACTIN2* (mean ± SE, *n* = 3). *P < 0.05, **P < 0.01 (Student’s *t*-test; mean ± SE, *n* = 3). **(F)** Trypan blue staining of leaves of WT Bu-5 and *aod12* seedlings under normal conditions and osmotic stress as in **(E)**.

In the Arabidopsis NER pathway, *UVH6*, along with *XPF*, *XPG*, and *ERCC1*, plays a role in damage-induced cleavage at the 5′ or 3′ ends (Tuteja et al., 2009; Yoshiyama et al., 2009). To further investigate the relationship between NER components and osmotolerance, we examined the sensitivity of the NER-related mutants (Col-0 background) *uvh6-1*, *xpf* (T-DNA insertion), *xpg* (strong allele), and *ercc1* (T-DNA insertion) to cisplatin-induced DNA damage stress. All mutants were hypersensitive to cisplatin, but sensitivity was greater in *xpf*, *xpg*, and *ercc1* than in *uvh6-1* (**Figure 7A**). However, *uvh6–1* had an osmosensitive phenotype, whereas *xpf*, *xpg*, and *ercc1* retained WT-like osmotolerance (**Figure 7B**). Among these mutants, only *uvh6–1* was hypersensitive to L-heat stress (**Supplementary Figure 2**). These results suggest that the decrease in osmotic and L-heat stress tolerance caused by the mutation in *UVH6* is independent of its DNA repair function.

### 3.4 *UVH6* mutation links osmotic stress response to immune activation

Given that *UVH6* has been implicated in heat stress–induced transcriptional changes and the release of heterochromatin silencing in Arabidopsis (Bourguet et al., 2018), we performed RNA-seq analysis of WT and *aod12* seedlings under normal and osmotic stress conditions, and identified differentially expressed genes (DEGs) (**Figure 7A** and **Supplementary Table 1**). Gene Ontology (GO) enrichment analysis revealed that osmotic stress response genes were enriched in WT-specific DEGs (**Figure 7B**), suggesting a partial reduction in osmotic stress response at the transcriptional level in *aod12*. *ORG1*, *WRKY54*, and *UGT71B6* belong to the GO term of osmotic stress response, and qRT-PCR confirmed that they were expressed at lower levels in *aod12* than in WT under osmotic stress (**Figure 7D**). For *aod12*-specific DEGs, GO terms related to decreased oxygen levels and defense responses were enriched (**Figure 7C**). To determine whether the immune response is activated in *aod12* plants, we analyzed the transcript levels of *pathogenesis-related* (*PR*) genes (*PR1*, *PR2*, and *PR5*) in *aod12* under osmotic stress. To further illustrate the altered gene expression in *aod12*, heatmaps of genes belonging to the “response to stress” and “defense response” GO terms, as identified in **Figure 7C**, are presented in **Supplementary Figures 3A, B**, respectively. The *PR* transcript levels were significantly higher in *aod12* than in WT under osmotic stress, in line with increased cell death (**Figures 7E, F**).

*UVH6* is required for efficient transcription at numerous loci under heat stress, as *uvh6* mutants exhibit reduced transcript accumulation at genome-wide loci, including heterochromatin regions, under heat stress (Bourguet et al., 2018). We compared relative transcript accumulation at the chromosomal level between *aod12* and WT under normal and osmotic stress conditions. Transcript accumulation was slightly higher in *aod12* than in WT across the genome (log2 fold change < 0.5) under normal conditions (**Supplementary Figure 4**), and did not differ significantly under osmotic stress (**Supplementary Figure 4**).

**Figure 8:**
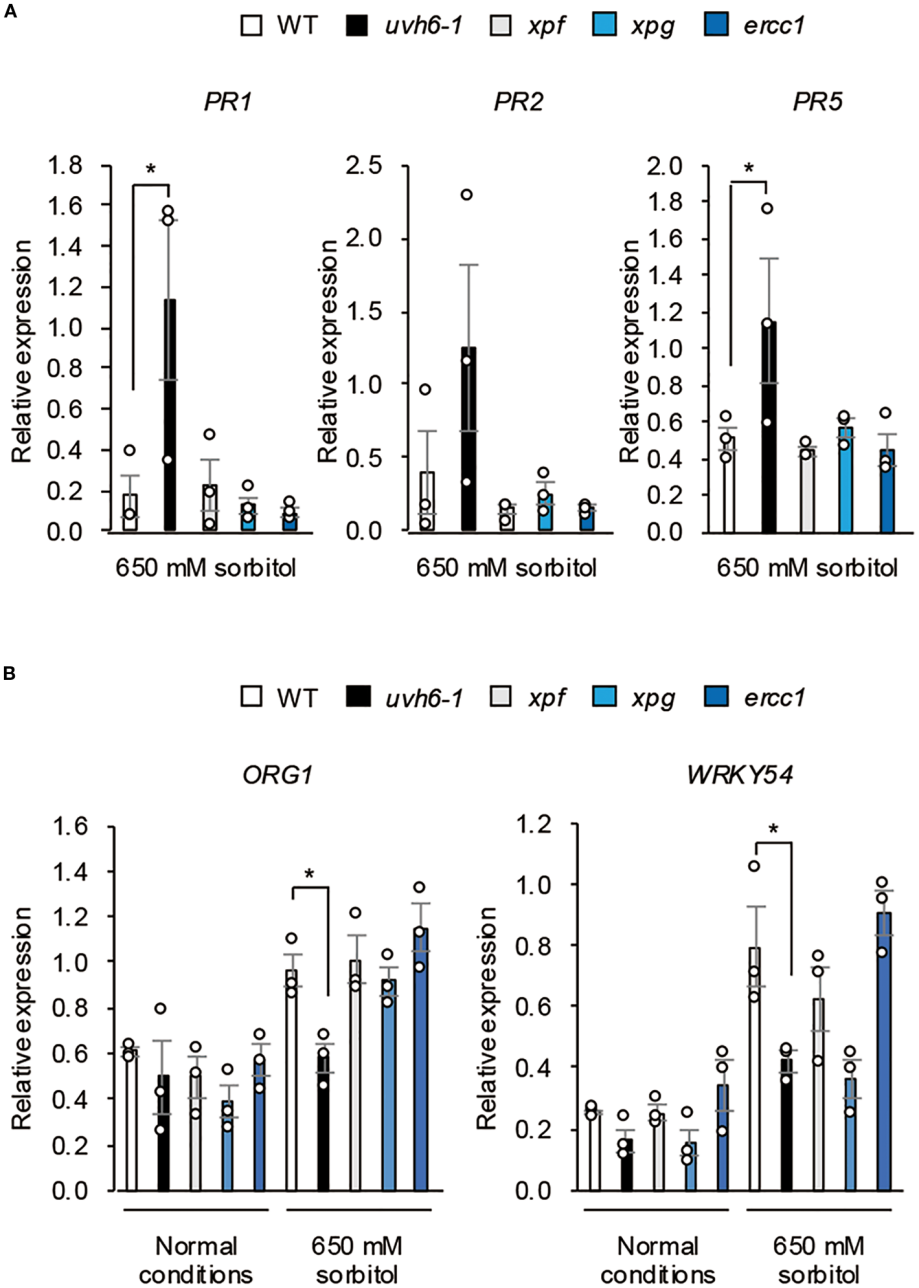
Effect of osmotic stress on gene expression in NER-related mutants. Expression of **(A)** osmotic stress–responsive genes and **(B)** *PR* genes in WT Col-0 and NER-related mutants (Col-0 background) under normal conditions and osmotic stress (100 mM NaCl for 7 days followed by 650 mM sorbitol for **(A)** 8 h or **(B)** 3 days). Transcript levels were determined by quantitative real-time PCR relative to those of *Actin2* (mean ± SE, *n* = 3). *P < 0.05 (Student’s *t*-test).

To determine whether the reduced osmotolerance of *uvh6* mutants was due to an enhanced immune response or impaired DNA repair, we analyzed *PR* gene expression under osmotic stress in the NER-related mutants (Col-0 background) *uvh6-1*, *xpf*, *xpg*, and *ercc1*. Under osmotic stress, the *PR* transcript levels were upregulated only in the *uvh6–1* mutant (**Figure 8A**) and the *ORG1* and *WRKY54* transcript levels increased or tended to increase in WT and all mutants except *uvh6-1* (for *ORG1*) or except *uvh6–1* and *xpg* (for *WRKY54*) (**Figure 8B**). These results suggest that the impaired osmotolerance in *UVH6* mutants is associated with an enhanced immune response and reduced expression of stress-response genes rather than defects in DNA repair.

While the precise molecular mechanism by which UVH6 regulates transcriptional responses and immune activation under osmotic stress remains to be fully elucidated, we sought to investigate the involvement of key immune regulators in the UVH6-mediated immune response. Given that heightened immune responses mediated by EDS1/PAD4 are known to compromise osmotic tolerance (Ariga et al., 2017; Kanamori et al., 2023), we generated a *pad4 aod12* double mutant using genome editing to test if PAD4 is involved in the immune activation observed in *aod12* (**Supplementary Figure 5A**). Upon assessing the osmotic tolerance of the *pad4 aod12* double mutant, we found that it displayed a hypersensitive phenotype to osmotic stress comparable to the *aod12* single mutant, when compared to the wild type (**Supplementary Figure 5B**). This striking result suggests that the immune activation induced by the *UVH6* mutation under osmotic stress is mediated by a signaling pathway that is independent of PAD4.

Given that UVH6 is an essential component of the TFIIH complex, crucial for plant viability and involved in transcription initiation, we sought to investigate the potential structural and functional impact of the *aod12* and *uvh6–1* mutations on UVH6 protein. We first performed a multiple sequence alignment and found that the R296 residue, which is deleted in the aod12 mutant, is highly conserved across various species, including mammals and other plants (**Supplementary Figure 6A**), suggesting its functional importance. Next, we utilized AlphaFold3 to predict the three-dimensional structures of wild-type, *aod12* mutant, and *uvh6–1* mutant UVH6 proteins. To validate AlphaFold3’s predictive capability for *Arabidopsis* XPD (UVH6), we first generated its three-dimensional structure and compared it with previously reported crystal structures of human XPD, which is highly conserved across species from humans to archaea (Kokic et al., 2019). The predicted *Arabidopsis* XPD structure showed good agreement with the known human XPD crystal structures, suggesting that AlphaFold3 can reliably predict the three-dimensional structure of XPD (**Supplementary Figure 6B, C**). Subsequently, we predicted the three-dimensional structures of wild-type *Arabidopsis* UVH6, *aod12* mutant type, and *uvh6–1* mutant type using AlphaFold3. Our predictions revealed that the *aod12* mutant type, which has a one-amino acid deletion in its Arch domain, is predicted to adopt an aberrant tertiary structure in this domain compared to the wild type (**Supplementary Figure 6D**). Interestingly, although the *uvh6–1* mutant type carries a one-amino acid substitution (glycine to glutamate) in the Helicase domain 2 (Bourguet et al., 2018), not the Arch domain, it too is predicted to exhibit an abnormal tertiary structure in the Arch domain, similar to the *aod12* mutant type (**Supplementary Figure 6D**). The Arch domain is known to be a crucial interaction site with MAT1, a core component of TFIIH (Peissert et al., 2020). We hypothesize that the structural aberrations in the Arch domain in both *aod12* and *uvh6–1* mutants may hinder their proper interaction with MAT1, leading to an abnormal TFIIH complex structure and consequently impacting transcription.

### 3.5 *UVH6* mutation triggers immune response to osmotic stress independently of *UVR8* and *SOG1*

UV RESISTANCE LOCUS 8 (UVR8) is an UV-B receptor; PR 1 and 5 proteins are induced more rapidly and to a higher extent in *uvr8–1* mutant than in WT (Kliebenstein et al., 2002). To investigate whether the enhanced immune response under osmotic stress caused by the *UVH6* mutation is mediated by UVR8, we examined the osmotolerance of the *uvr8* mutant. Its osmotolerance was similar to that of WT, and it had no osmosensitive phenotype observed in *uvh6-1* **(Supplementary Figure 7A**). The L-heat tolerance of *uvh6–1* was also similar to that of WT (**Supplementary Figure 7B**), suggesting that the immune response triggered by the *UVH6* mutation under osmotic stress is independent of the UVR8 pathway.

Immune responses are activated by DNA damage via SUPPRESSOR OF GAMMA RESPONSE1 (SOG1) (Yoshiyama et al., 2020) Double mutations in *SOG1* and *XPF* suppress the hypersensitivity to gamma rays but do not affect the UV sensitivity observed in *XPF* single mutants (Preuss and Britt, 2003). To examine the role of SOG1 in UVH6-dependent osmotolerance, we generated a *uvh6–1 sog1–1* double mutant in the Col-0 background and compared its osmotolerance with that of WT, *uvh6-1*, and *sog1-1*. That of *sog1–1* was similar to that of WT, while the double mutant was hypersensitive to osmotic stress, similar to *uvh6-1* (**Supplementary Figure 7C**), suggesting that the immune response induced by the *UVH6* mutation under osmotic stress is independent of SOG1-mediated immune regulation. To further confirm this, we investigated the expression of the immune-related genes *PR1* and *PR2* under osmotic stress. As shown in **Supplementary Figure 7D**, the expression of both *PR1* and *PR2* was higher in the *uvh6–1* and *uvh6–1 sog1–1* double mutant compared to WT. Notably, the expression levels in the double mutant were even higher than those in the *uvh6–1* single mutant, indicating that the *sog1–1* mutation does not suppress the osmotic stress-induced expression of these immune genes. Collectively, these results indicate that the immune response pathway induced by the *UVH6* mutation under osmotic stress is independent of *UVR8* and *SOG1* signaling.

## 4 Discussion

Here we isolated the *aod12* mutant, which is hypersensitive to osmotic and heat stresses, and identified *UVH6* as the causative gene. The *uvh6* mutants are known to be highly sensitive to UV-B, UV-C and heat stress, but the role of *UVH6* in osmotolerance has not been reported. UVH6 is a component of the TFIIH complex, which functions as a transcription factor and a major component of NER, a key DNA repair pathway. We investigated whether the hypersensitivity to osmotic stress caused by *UVH6* mutations was attributable to either or both of these functions.

While both *aod12* and *uvh6–1* are *UVH6* mutants exhibiting compromised osmotic stress tolerance, distinct differences in their phenotypes, particularly concerning chlorophyll content, were observed. As evident in **Figure 1** and **3**, the *uvh6–1* mutant consistently displays significantly lower chlorophyll content and a more pronounced pale green phenotype compared to *aod12* under normal growth conditions, and a seemingly more severe reduction under osmotic stress. These phenotypic discrepancies likely reflect differences in the severity of the two *UVH6* alleles. Our data suggest that the aod12 mutation (R296 deletion) results in a weaker functional impairment of UVH6 compared to the *uvh6–1* allele. While both mutations affect UVH6 function, the more drastic impact on chlorophyll content in *uvh6–1* points to a more severe disruption of UVH6’s role in processes related to chloroplast development or maintenance. It is also important to acknowledge that the distinct genetic backgrounds of *aod12* (Bu-5) and *uvh6-1* (Col-0) could contribute to the observed phenotypic variations, making direct quantitative comparisons of osmotic tolerance between the two alleles challenging.

We examined the relationship between osmotic stress and DNA damage in *Arabidopsis* and found that osmotic stress induces DNA damage, and that pretreatment with cisplatin reduces osmotolerance, collectively suggesting that DNA repair is crucial in the osmotic stress response (**Figures 4B**, **5**). The *aod12* mutant exhibits hypersensitivity to cisplatin (**Figure 4A**), a known inducer of DNA cross-links, indicating impaired DNA repair function. This is consistent with UVH6’s established role in Nucleotide Excision Repair (NER).

However, a key observation that initially appears paradoxical is that despite the impaired NER function in *aod12*, the extent of osmotic stress-induced DNA damage (both single and double-strand breaks) was comparable between WT and *aod12* (**Figure 2**). This suggests that while osmotic stress indeed causes DNA damage and DNA repair is important, the specific contribution of UVH6’s NER activity may not be the primary factor determining the overall level of DNA damage under osmotic stress, or other repair pathways might largely compensate for this particular type of damage.

Furthermore, a critical distinction emerged when comparing *uvh6–1* with other NER factor mutants. While the NER factor mutants *uvh6-1, xpf, xpg*, and *ercc1* all shared hypersensitivity to cisplatin, only *uvh6–1* was hypersensitive to osmotic and heat stresses (**Figure 7B**). This strongly suggests that the decrease in osmotolerance and heat tolerance caused by the UVH6 mutation is not solely dependent on its canonical DNA repair function as part of NER, as other NER mutants do not exhibit these stress sensitivities. Instead, these findings collectively lead us to propose that while DNA damage repair is essential for osmotic stress tolerance in *Arabidopsis*, and UVH6 contributes to NER, UVH6’s unique and more prominent role in mediating osmotic and heat stress tolerance likely lies in its function in transcription, a role that distinguishes it from other NER components in this context.

Comparison of DEGs under osmotic stress between WT and *aod12* revealed that the transcript levels of certain osmotic stress–responsive genes were lower in *aod12* than in WT, indicating that UVH6 is involved in the transcriptional response to osmotic stress. Among the *aod12*-specific osmotic stress–induced DEGs, genes associated with response to oxygen-containing compound and hypoxia response were enriched. These GO terms suggest that oxidative stress is enhanced in *aod12* under osmotic stress. The enhanced oxidative damage response in *aod12* may explain why NER’s DNA repair function does not sufficiently contribute to osmotolerance, but is important for suppressing oxidative stress caused by osmotic stress. We would like to clarify that while the *uvh6* mutation leads to a global reduction in transcription across many gene loci under heat stress (Bourguet et al., 2018), its transcriptional effects under osmotic stress are more limited. As shown in Supplementary **Figure 5**, we did not observe the genome-wide reduction in gene expression under osmotic stress that is characteristic of *uvh6* under heat stress. This suggests that under osmotic stress, UVH6’s role in transcriptional regulation is restricted to specific gene subsets, rather than exhibiting broader, global effects.

While previous reports indicate that the heat-induced accumulation of canonical heat-responsive factors like HSFs and HSPs is independent of UVH6 (Larkindale et al., 2005; Hu et al., 2015), reinforcing the notion that UVH6 is not universally required for the transcription of all genes during heat stress, our current findings suggest that UVH6 does influence the expression of at least a subset of *HSP* genes. The discrepancy might arise from differences in heat treatment duration or the specific *uvh6* mutations examined, highlighting the nuanced role of UVH6 in the heat stress response.

A key finding of our study is the distinct phenotypic and molecular responses of *uvh6* mutants compared to other NER-deficient mutants. While the *uvh6-1*, *xpf*, *xpg*, and *ercc1* mutants all shared hypersensitivity to the DNA-damaging agent cisplatin, only the *uvh6–1* mutant exhibited high sensitivity to osmotic stress (and heat stress) compared to WT. This suggests that the reduced stress tolerance in *uvh6* is not a general consequence of a defect in NER.

A major mechanistic basis for this distinction lies in the immune response pathway. As we have demonstrated, the *aod12* mutant had an enhanced immune response under osmotic stress accompanied by increased leaf cell death. This is a critical factor, as enhanced immune responses are known to suppress osmotolerance (Ariga et al., 2017; Uchida et al., 2022; Kanamori et al., 2023; Murakoshi et al., 2024). Critically, this enhanced immune response was not observed in the *xpf*, *xpg*, or *ercc1* mutants under the same conditions. This finding is central to our argument: it suggests that UVH6 uniquely regulates immune activation, and this function is independent of its canonical DNA repair role within the NER pathway. We therefore propose that this non-canonical function of UVH6 in modulating the immune response is a major contributor to its osmosensitive phenotype, distinguishing it from other NER components.

DNA damage activates immune responses via SOG1 (Preuss and Britt, 2003; Yoshiyama et al., 2020), but the osmotolerance of the *sog1* mutant and WT was comparable, and the *uvh6–1 sog1* double mutant retained the osmosensitive phenotype of *uvh6-1*. Therefore, the immune response pathway induced by the *UVH6* mutation under osmotic stress is independent of SOG1 signaling. The receptor for UV-B UVR8 regulates immune responses under UV stress (Kliebenstein et al., 2002), but the osmotolerance of the *uvr8–1* mutant and WT was comparable. Therefore, the osmosensitive phenotype of *aod12* is independent of UVR8 signaling. A mitogen-activated protein kinase (MAPK) pathway is involved in UV stress tolerance independently of UVR8 (Ulm et al., 2002; Kalbina and Strid, 2006; Besteiro et al., 2011; Besteiro and Ulm, 2013). The *mkp1* mutant, lacking *MAPK phosphatase 1* (*MKP1*), is highly sensitive to UV-B stress. Notably, *MKP1* is the causal gene in *aod13*, another mutant hypersensitive to osmotic stress (Uchida et al., 2022). In *aod13*, *PR* gene expression is increased under osmotic stress (Uchida et al., 2022). However, further genetic analysis is required to clarify the potential overlap between the UVH6 and MKP1 pathways in osmotolerance. Interestingly, *aod13* is not particularly heat sensitive; therefore, the heat-sensitive phenotype of *aod12* is independent of the MKP1 pathway. Our study revealed that both *aod13* and *aod12* mutants display compromised osmotolerance due to an enhanced immune response. However, we found a crucial difference in their heat stress tolerance. To investigate the mechanistic basis for this distinction, we examined the expression of *heat shock protein* (*HSP*) genes in *aod12* under heat stress conditions. As shown in **Figure 2D**, the expression levels of both *HSP70* and *HSP17.6* were significantly lower in *aod12* compared to WT when subjected to heat stress. This attenuated HSP induction suggests that UVH6 influences the transcription of at least a subset of these key stress-responsive genes, and its impaired function likely contributes to the heat hypersensitivity of *aod12*. These findings, combined with previous reports that UVH6 has a unique role in genome-wide transcriptional regulation under heat stress (Bourguet et al., 2018), indicate that while both *aod12* and *aod13* share a defect in osmotolerance, the heat-sensitive phenotype is specific to *aod12*, likely due to UVH6’s distinct transcriptional role under heat stress that is not affected by the *aod13* mutation.

Here, we established that UVH6 is critical for osmotic and heat stress tolerance, and that these functions are independent of its canonical DNA repair function. Our findings indicate that UVH6 regulates transcriptional responses under osmotic stress, and its mutation leads to aberrant immune activation and cell death, ultimately compromising stress resilience. These findings provide new insights into the role of *UVH6* in osmotic stress response, linking it to immune activation rather than DNA repair.

## Data Availability

The datasets presented in this study can be found in online repositories. The names of the repository/repositories and accession number(s) can be found in the article/**Supplementary Material**.

## Supplementary material

Supplementary Figure 1 Complementation test. T_2_ plants (*aod12*_*UVH6*) were obtained after complementation of *aod12* with *UVH6* (*At1g03190*) under the control of its native promoter from WT Bu-5. Images of WT Bu-5, *aod12*, and *aod12*_*UVH6* plants grown under normal conditions and their chlorophyll content are shown. The same letters above the bars indicate no significant differences (*P* < 0.05, one-way ANOVA with *post hoc* Tukey HSD test; mean ± SE, *n* = 3).

Supplementary Figure 2 L-heat tolerance of NER-related mutants (Col-0 background).Ten-day-old seedlings were placed at 37°C for 4 days and then returned to 22°C for 7 days.

Supplementary Figure 3 Heat map of log2 fold change in gene expression of *aod12* belonging to GO terms “stress response **(A)**” and “defense response **(B)**” identified in **Figure 1C**.

Supplementary Figure 4 Transcriptomic analysis of *aod12*. Transcriptional changes in *aod12* seedlings relative to WT Bu-5 under **(A)** normal conditions and **(B)** osmotic stress (100 mM NaCl for 7 days followed by 750 mM sorbitol for 8 h). RPKM, reads per kilobase per million mapped reads; the values were calculated in 100 kb windows.

Supplementary Figure 5 Role of TIR-NLR immune regulator PAD4 in uvh6 mutation-induced immune response under osmotic stress. **(A)** Editing of the *PAD4* gene in *aod12* by the CRISPR/Cas9 system. The top panel shows that the gRNA was designed to target the sixth exon, and two independent mutant lines, *pad4 aod12 <ns/>1* and *pad4 aod12 <ns/>2*, were obtained. The bottom panel shows a sequence comparison of the WT, *pad4 aod12 <ns/>1*, and *pad4 aod12 <ns/>2*. The *pad4 aod12 <ns/>1* mutant has an 8-bp deletion, while *pad4 aod12 <ns/>2* has a 1-bp (G) insertion. As a result, premature stop codons were introduced at the 263rd amino acid in *pad4 aod12 <ns/>1* and at the 265th amino acid in *pad4 aod12 <ns/>2*, suggesting the truncation of functional PAD4 protein. **(B)** Upper photos: 14-day-old WT, *aod12*, *pad4 aod12_<ns/>1* and *pad4 aod12_<ns/>2* seedlings grown under normal conditions. Lower photos: Acquired osmotolerance of WT, *aod12*, *pad4 aod12_<ns/>1* and *pad4 aod12_<ns/>2*. Salt-acclimatized two-week-old seedlings were mesh-transferred to MS agar plates containing 700 mM sorbitol for 10 d.

Supplementary Figure 6 Protein structure prediction of UVH6. **(A)** Multiple sequence alignment of XPD/UVH6 protein. The partial amino acid sequences of XPD from *Arabidopsis thaliana*, *Oryza sativa* (rice), *Physcomitrium patens* (moss), *Mus musculus* (mouse), and *Homo sapiens* (human) are shown. The position corresponding to the deleted Arginine residue (R296) in the *aod12* mutant is highlighted in red. Asterisks (*) indicate conserved amino acid residues. This alignment demonstrates that the R296 residue is highly conserved across diverse species. **(B)** The predicted structure of UVH6. **(C)** Superposition of UVH6 (Arabidopsis XPD) and human XPD. (**D**) The crystal structures of wild-type Arabidopsis UVH6, aod12 mutant type, and uvh6–1 mutant type. The predicted structure of UVH6 colored according to the pLDDT values [blue (high) to yellow (low)].

Supplementary Figure 7 Osmotic stress tolerance of mutants defective in DNA damage and immune responses. **(A)** Acquired osmotolerance of *uvr8*. Salt-acclimated 2-week-old seedlings of WT Col-0 and *uvr8* mutant (Col-0 background) were mesh-transferred to MS agar plates containing 650 mM sorbitol for 18 days. **(B)** L-heat tolerance of *uvr8*. Ten-day-old WT and *uvr8* seedlings grown at 22°C (left image) were placed at 37°C for 4 days and then returned to 22°C for 7 days (right image). **(C)** Acquired osmotolerance of WT, *uvh6-1*, *sog1-1*, and *uvh6–1 sog1-1* (Col-0 background). Stress was applied as in **(A, D)** Expression of *PR* genes in WT, *uvh6-1*, *sog1-1*, and *uvh6–1 sog1-1* (Col-0 background) under normal conditions and osmotic stress (100 mM NaCl for 7 days followed by 650 mM sorbitol for 3 days). Transcript levels were determined by quantitative real-time PCR relative to those of *Actin2* (mean ± SE, *n* = 3). **P* < 0.05, ****P* < 0.001 (Student’s *t*-test).
