# Supplementary material for: UVH6 regulates osmotic and heat stress tolerance by modulating transcription
Source: Front Plant Sci. 2025 Sep 18;16:1623563. doi: 10.3389/fpls.2025.1623563 (PMC12504840; doi:10.3389/fpls.2025.1623563)
Supplement: Supplementary Figure 1 — Complementation test. T2 plants (aod12_UVH6) were obtained after complementation of aod12 with UVH6 (At1g03190) under the control of its native promoter from WT Bu-5. Images of WT Bu-5, aod12, and aod12_UVH6 plants grown under normal conditions and their chlorophyll content are shown. The same letters above the bars indicate no significant differences (P < 0.05, one-way ANOVA with post hoc Tukey HSD test; mean ± SE, n = 3). [file DataSheet1.pdf]

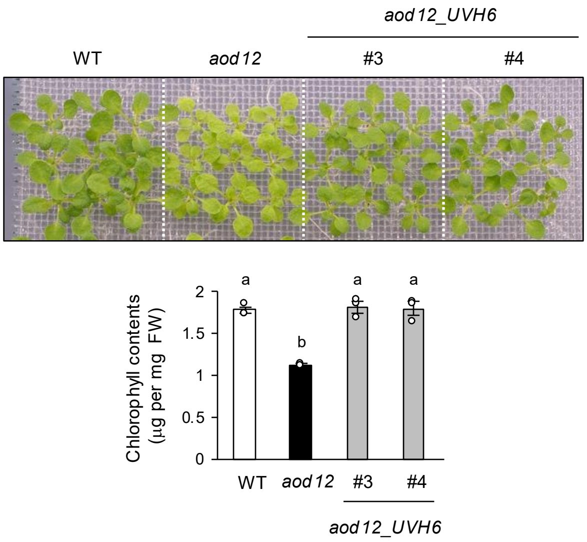

Supplementary Figure 1

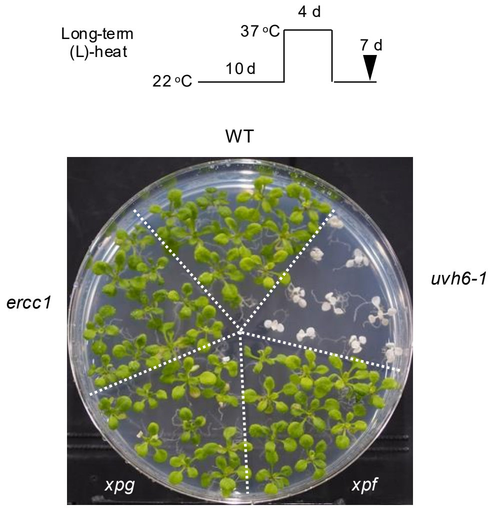

Supplementary Figure 2

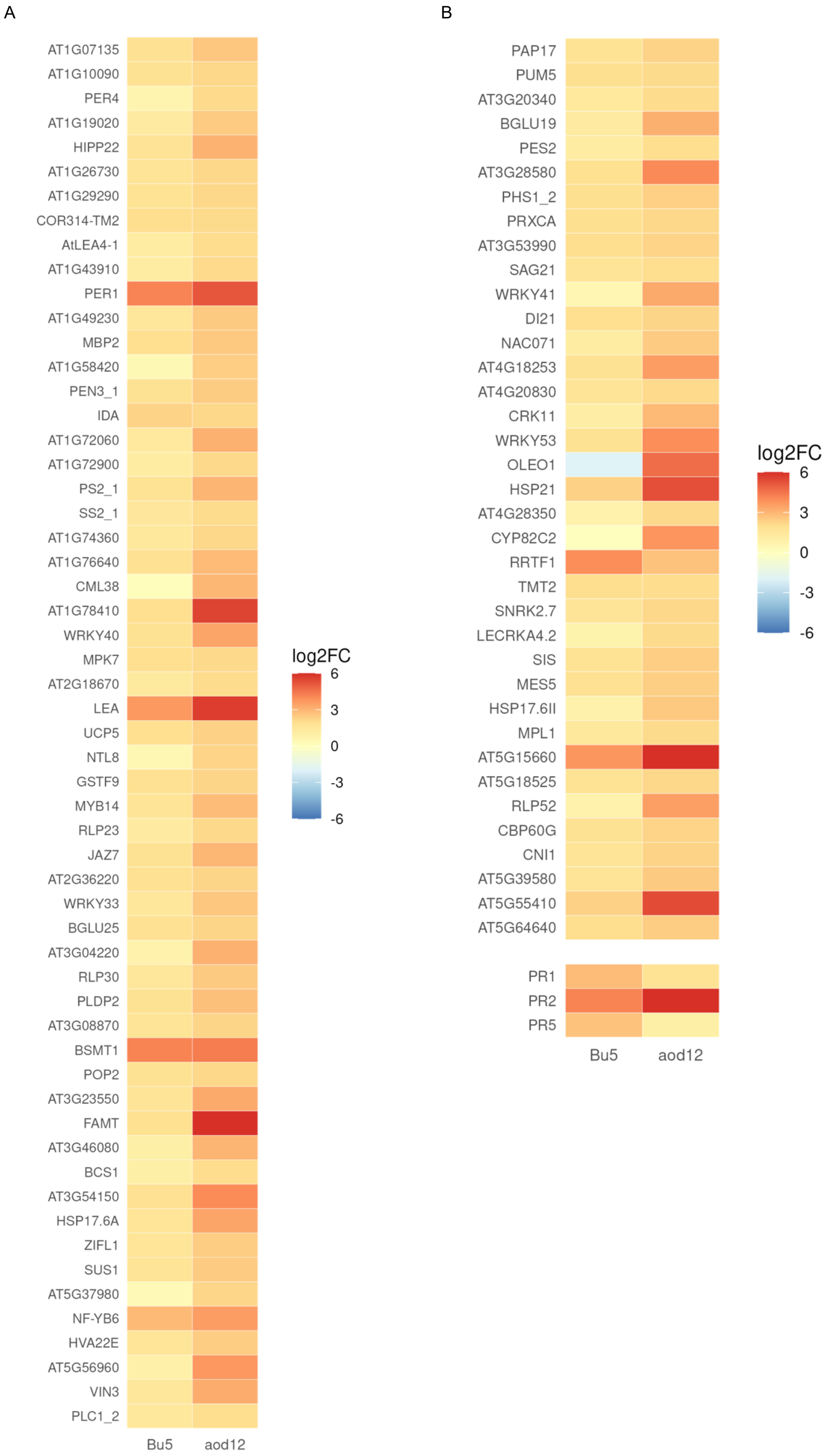

Supplementary Figure 3

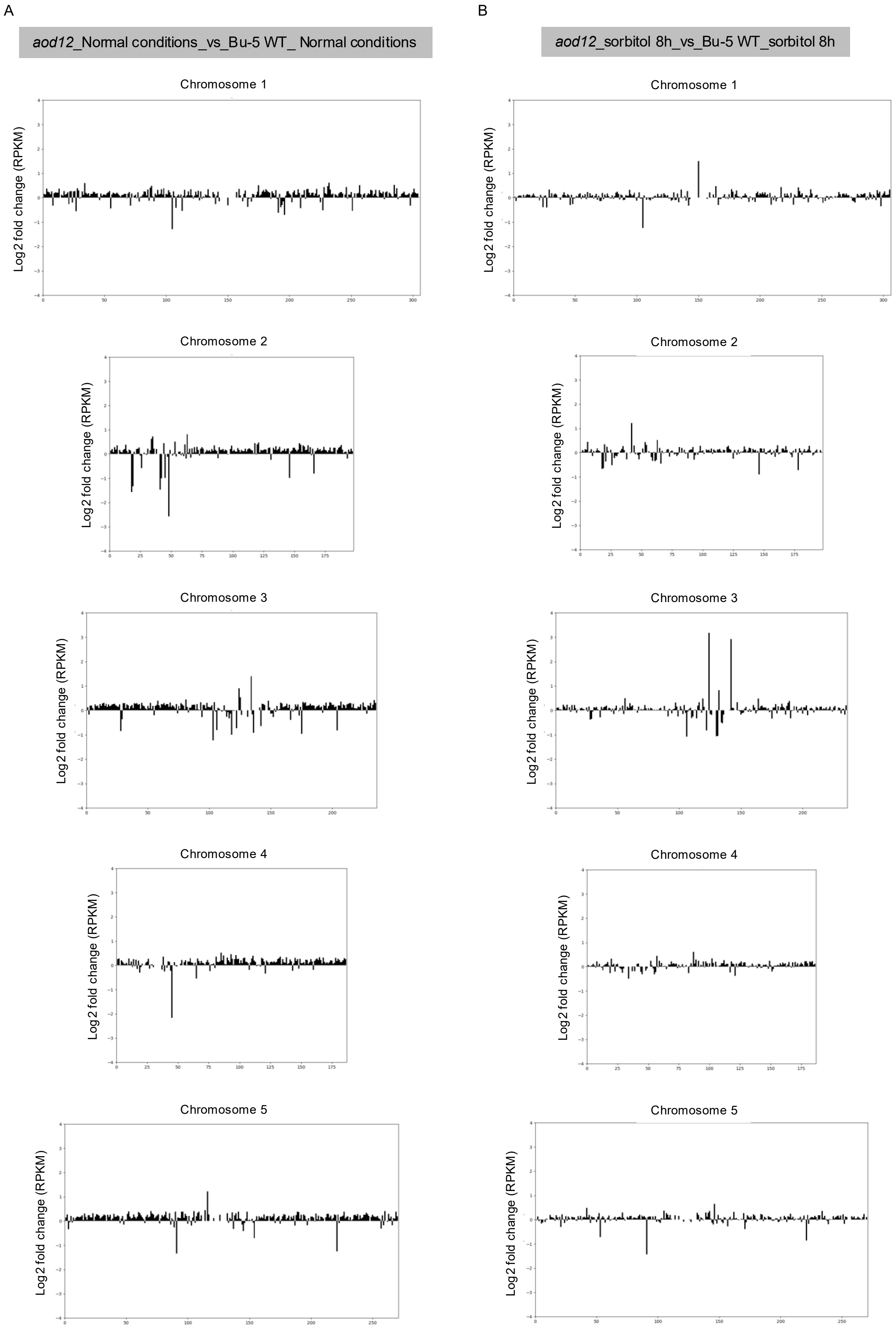

Supplementary Figure 4

A

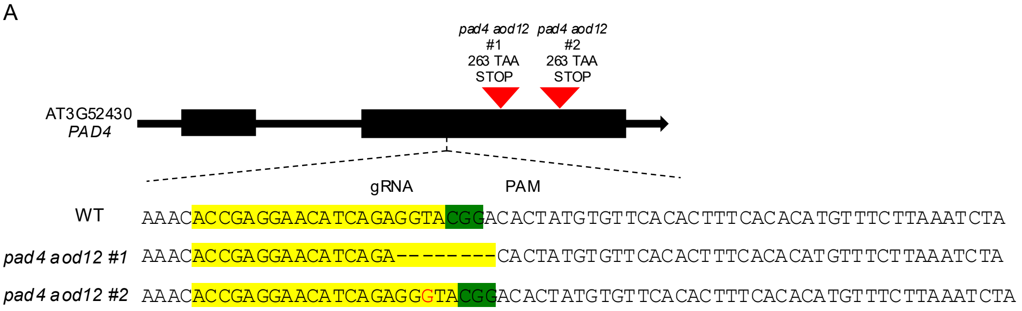

B

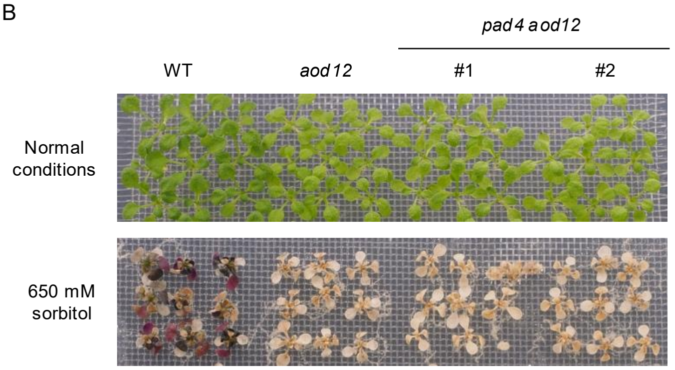

Supplementary Figure 5

A

```
Arabidopsis thaliana XPD/UVH6   IRQEIDRFKATDAGRLRAEYNRLVEGLALRGDLSGGDQWLA
Oriza sativa XPD                ISQEIDRFKATDANRLRAEYNRLVDGLAQRGNLPISDAWLA
Physcomitrium patents XPD       VTQLIEKSKATDANRLRQEYSRLVEGLAQRGNLAGTDTWLA
Mus musculus XPD                LQKTVLRIKETDEQRLRDEYRRLVEGLREASVARETDAHLA
Homo sapiens XPD                LQKTVLRIKETDEQRLRDEYRRLVEGLREASAARETDAHLA
                                        * **  *** ** *** **      *  *  **
```

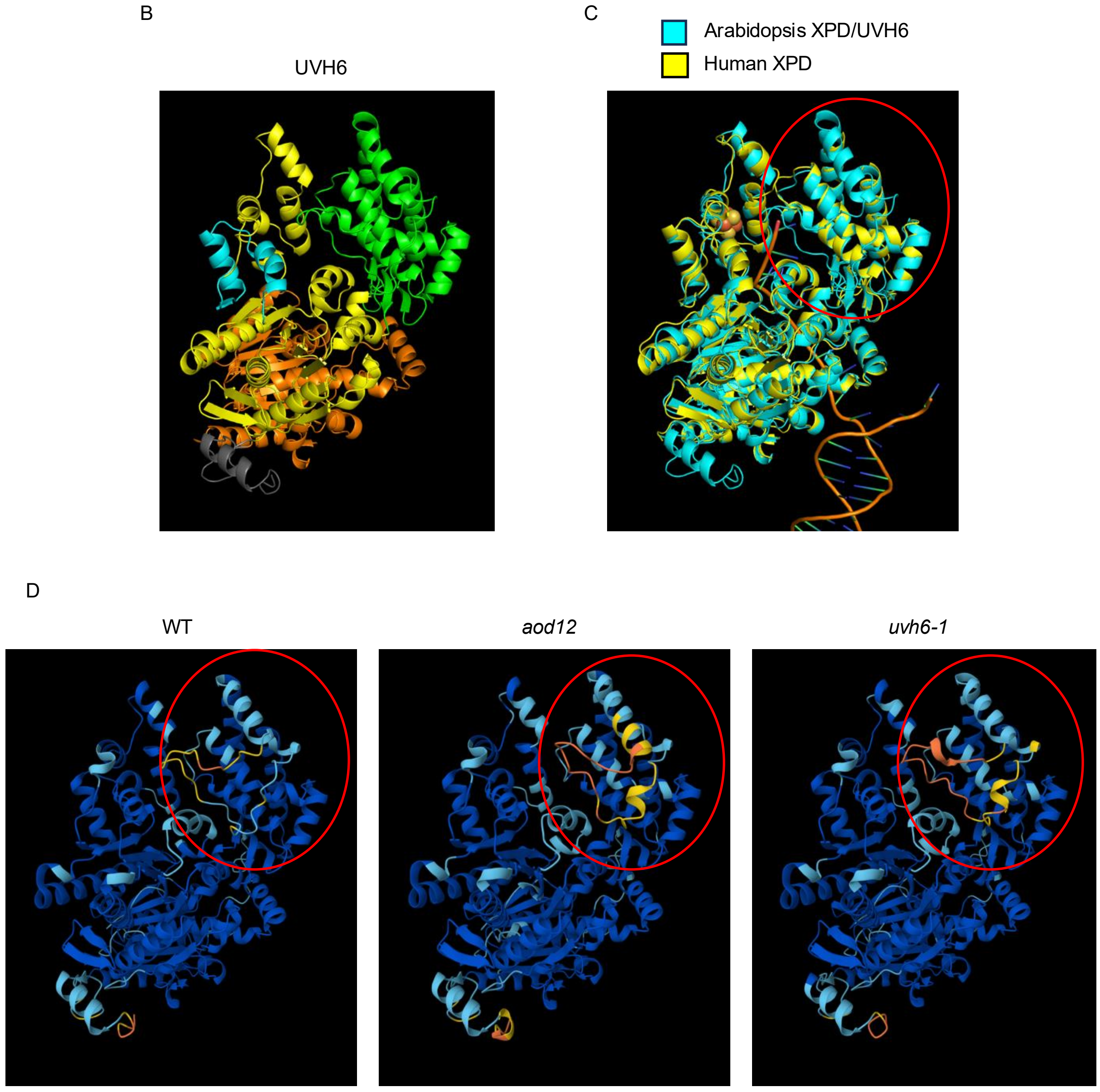

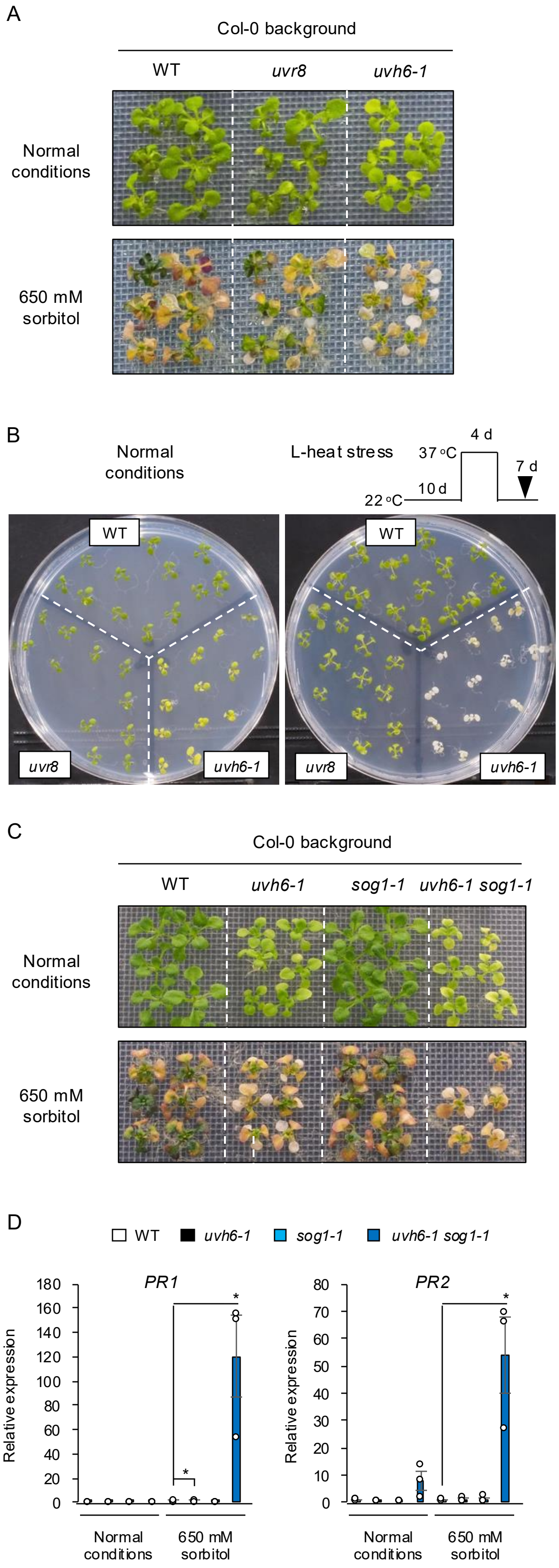

Supplementary Figure 7
